# Financial Strain, Food Insecurity, and Spiritual Beliefs on Depression in Middle-Aged and Older Indigenous Women

**DOI:** 10.1093/geroni/igaf122.3051

**Published:** 2025-12-31

**Authors:** Soonhee Roh, Yeon-Shim Lee, Erna Dinata, Sukyung Yoon, Joel Steele, Elizabeth Peffer Talbot, Sasheen T Stone

**Affiliations:** University of South Dakota, Sioux Falls, South Dakota, United States; San Francisco State University, San Francisco, California, United States; University of South Dakota, Sioux Falls, South Dakota, United States; University of North Texas, Denton, Texas, United States; University of North Dakota, Grand Forks, North Dakota, United States; Wright State University, Dayton, Ohio, United States; Yankton Sioux Tribe, Wagner, South Dakota, United States

## Abstract

**Purpose:**

This study investigates how financial strain and food insecurity contribute to depression among middle-aged and older Indigenous women. It further explores whether religious and spiritual beliefs serve as a buffer against the impact of these stressors, providing insight into their potential protective role in mental health.

**Method:**

In collaboration with a Northern Plains tribe, this study included 133 self-identified Indigenous women aged 40 to 70 years. The stress process model (SPM) provided the theoretical framework. Standardized assessments, including the Patient Health Questionnaire (PHQ-9), measures of food insecurity, financial strain, and religious/spiritual beliefs, were administered. Path analysis was used to assess both direct and indirect effects.

**Results:**

The study found a significant burden of depression, with 19.5% of participants experiencing mild depression, 11.3% moderate depression, 5.3% moderately severe depression, and 3% severe depression. Results support the SPM, indicating that financial strain and food insecurity are significantly associated with depressive symptoms. Financial strain influenced depression indirectly through its association with food insecurity. However, religious and spiritual beliefs did not act as protective factors against depressive symptoms in this population.

**Conclusion:**

This study underscores the complex interplay of financial strain, food insecurity, and depressive symptoms among middle-aged and older Indigenous women. Findings highlight the need for targeted, multidimensional interventions addressing both economic and psychosocial factors to improve mental health outcomes in this population. Future research should refine strategies to enhance well-being and resilience.

